# The Effectiveness of Mental Health Literacy Curriculum among Undergraduate Public Health Students

**DOI:** 10.3390/ijerph19095269

**Published:** 2022-04-26

**Authors:** Hsuan-Jung Lai, Yin-Ju Lien, Kai-Ren Chen, Yu-Kai Lin

**Affiliations:** 1 Department of Health Promotion and Health Education, National Taiwan Normal University, 162, Heping East Road Section 1, Taipei 106, Taiwan; jessie052909@gmail.com; 2 Department of Public Health, Fu Jen Catholic University, 510, Zhongzheng Road, New Taipei 242, Taiwan; ph1010@mail.fju.edu.tw; 3 Department of Health and Welfare, University of Taipei, 101, Zhongcheng Road Section 2, Taipei 111, Taiwan; yklin@utaipei.edu.tw

**Keywords:** mental health literacy, curriculum-based intervention, school-based program, public health education, university students

## Abstract

Mental health literacy (MHL) plays an important role in public health. Improving MHL can promote mental health at the individual and public levels. To date, no published studies have assessed the effectiveness of MHL curriculum interventions among undergraduate public health students. The participants in this study were undergraduate public health students (*n* = 48) who were enrolled in an 18-week MHL curriculum for 100 min per week. MHL was assessed using the Mental Health Literacy Scale for Healthcare Students. A paired sample *t*-test was performed to examine the immediate and delayed effects of the MHL curriculum. The total MHL score significantly improved, and a moderate effect size was found directly after the intervention and six weeks later. There were significant differences in the recognition of mental illness (*p* < 0.01), help-seeking efficacy (*p* < 0.05), and help-seeking attitude (*p* < 0.05) in the five components of MHL between pre- and post-test. Furthermore, significant improvements were obtained for the maintenance of positive mental health (*p* < 0.05) and reduction of mental illness stigma (*p* < 0.001) between the pre-test and follow-up. Our findings provide evidence for the development and implementation of an MHL curriculum for public health education.

## 1. Introduction

Jorm et al. [1] first defined mental health literacy (MHL) as “knowledge and beliefs about mental disorders which aid their recognition, management or prevention”. Jorm [2] then extended the concept of MHL into five elements: (1) understanding how to prevent mental illness; (2) understanding when a disorder is developing; (3) awareness of support and treatments for mental illness, (4) ability to effectively address mild mental health problems, and (5) mental health first aid skills to support others. Recently, Kutcher, Wei, and Coniglio [3] unified and expanded the definition of MHL. They proposed four components, namely: (1) knowledge about obtaining and maintaining good mental health; (2) understanding mental illnesses and treatments; (3) reducing mental illness-related stigma; and (4) enhancing help-seeking efficacy. Based on the definitions proposed by Jorm [2] and Kutcher et al. [3], Chao et al. [4] added help-seeking attitude as a component of MHL, because it was found to be a powerful predictor of help-seeking behavior [5].

A study investigating the prevalence of mental illness among university students found that the lifetime prevalence of at least one mental illness was 35.3%, and the 12-month prevalence was 31.4% [6]. During the COVID-19 pandemic, 71% of university students reported an increase in stress and anxiety [7]. Research has shown that 86% of undergraduates with mental health problems will drop out of school [8]. Despite the high prevalence of mental illness among university students, less than half seek professional help [9]. Possible reasons are poor MHL, such as inadequate positive mental health (i.e., the ability to manage stress or emotion) [10], lack of knowledge about mental illness [11,12], stigma associated with mental illness [12,13], and not understanding how to seek help [12,14]. Accordingly, MHL among university students must be improved.

Public health education aims to cultivate public health professionals in this respect [15,16]. Healthcare professionals are experiencing huge clinical care workloads after the COVID-19 outbreak, which has placed a heavy burden on their physical and mental health [17,18,19]. In addition to healthcare professionals, public health professionals are also under significant pressure due to outbreak investigations and epidemic management. A study in Taiwan indicated that work burnout is prevalent among public health staff; thus, mental health promotion or education programs are important for this group [20]. If mental health promotion and education can be started earlier during the university period, it can prevent public health students from being unable to complete their studies because of mental health problems. Moreover, it may help them detect mental health problems early, know how to seek help, and face these issues with a positive attitude in the event of a mental health crisis, which may ultimately promote their own mental health.

To date, interventions for MHL among university students have targeted medical, nursing, and pharmacology students [21,22,23,24]. In public health courses, curricula related to MHL cover mental health and general psychology. However, these are basic curricula, comprising just two hours per week for a single semester. To our knowledge, there has been no published research on the effects of MHL interventions on undergraduates in public health to date. Therefore, this intervention study aimed to improve MHL among undergraduate public health students. We hypothesized that students would demonstrate superior MHL after completing the MHL curriculum.

## 2. Methods

### 2.1. Participants

The inclusion criteria for participation were current enrollment in an undergraduate program in a public health-related department, and aged 18 years old or over. The participants were at two universities in the Taipei Metro Area. This study was conducted using convenience sampling between September 2020 and February 2021. The evaluation involved a one-group pretest-posttest design. Before and after the study, the students were asked to fill out a questionnaire in class. It took approximately 10 min to complete. In total, 60 students participated in the MHL curriculum on their own, but only 48 completed the pre-test, post-test, and six-week follow-up, an 80% response rate. To encourage participation, participants were compensated around NT$ 150 after completing the surveys.

To confirm that the sample size in the study had appropriate statistical power, G*Power 3.1 (Heinrich-Heine-Universität, Düsseldorf, Germany) was used to calculate the sample size [25]. The results indicated that 34 participants would be required, where α = 0.5, β = 0.2, assuming a moderate effect size (d = 0.5). The data of 12 people were missing more than 10% of the overall data; thus, these were regarded as invalid and were deleted [26]. As mentioned, 48 participants completed three valid surveys. Therefore, the sample size is adequate.

### 2.2. Intervention

We used content analysis, modified Delphi method and a focus group interview to develop the curricula modules of MHL in public education. In order to construct a curriculum with the five major components of MHL, the present research reviewed previous studies and research projects to develop a modified Delphi questionnaire. A panel of experts and scholars were invited to participate in the modified Delphi process. After two rounds of modified Delphi surveys, fourteen MHL learning objectives and sixty-eight learning components were constructed. On the basis of these findings, an 18-week MHL curriculum was developed which was thoroughly evaluated by means of a student focus group and an expert focus group. This intervention was an 18-week MHL curriculum of 100 min per week. It consisted of four main sections with a variety of delivery modes: (1) MHL lectures delivered in elective courses by the two professors specializing in mental health and with experience in the field; (2) people with mental illnesses were invited to discuss their mental status and recovery, and to answer students’ questions about mental illness; (3) films or short videos providing students with mental health-related knowledge and experience; and (4) group presentations requiring students to cooperate with each other and learn more about MHL-related issues. The curriculum materials included PowerPoint presentations, supplementary videos, and books related to MHL. The MHL curriculum contained several components (see Table 1) including:

1. Maintenance of positive mental health: Students acquire positive mental health-related concepts and skills by learning about self-concept, self-esteem, self-efficacy, and self-regulation; human relationships and communication skills; and stress and emotion management.
2. Recognition of mental illness and reduction of stigma: Students obtain knowledge regarding the symptoms, epidemiology, prevention, and treatment of common mental disorders (i.e., depression, anxiety, schizophrenia), and can identify negative attitudes toward mental illness.
3. Help-seeking efficacy and attitude: Students obtain knowledge regarding improving their own and others’ help-seeking resources, skills, and attitudes.

**Table 1:** MHL curriculum components, target concepts and delivery mode.

MHL Components	Target Concepts	Delivery Mode
Maintenance of positive mental health	Self-concept, self-esteem, self-efficacy and self-regulation, human relationships and communication skills, stress and emotion management	Lecture, cooperative learning
Recognition of mental illness	Mental illness and global mental health review, depression, bipolar disorder, anxiety disorder, schizophrenia, suicide and self-mutilation	Lecture, direct contact (face-to-face), indirect contact (film based), cooperative learning
Reduction of stigma associated with mental illness	The impact of mental illness stigma, strategies for stigma reduction	Lecture, direct contact (face-to-face), indirect contact (film based), cooperative learning
Recognition of mental illness, reduction of stigma associated with mental illness	To understand mental illness and reduce mental illness stigma by visiting mental rehabilitation institutions and watching a video related to mental illness and the process of rehabilitation	Lecture, direct contact (face-to-face), indirect contact (film based), cooperative learning
Help-seeking efficacy, help-seeking attitude	Resources of mental health care, help-seeking and helping skills	Lecture, indirect contact (film based), cooperative learning

### 2.3. Measures

#### 2.3.1. Demographics

Participants were asked to complete a demographics questionnaire that included items on gender, age, and living situation (i.e., with family, with relatives, with roommates, with friends, or alone).

#### 2.3.2. Level of Contact Report

The revised version of the Level of Contact Report [LCR] was developed by Corrigan et al. [27] to assess familiarity with mental illness. It consists of eight options regarding people’s intimacy with people with mental illness, ranging from low intimacy (“I have never observed a person that I was aware had a mental illness”) to high intimacy (“I have a mental illness”). Scores range from 0–7, with higher scores indicating higher familiarity with mental illness. Previous studies have confirmed the interrater reliability and validity of this instrument [28,29].

#### 2.3.3. Positive Mental Health Scale

The five-item revised Positive Mental Health Scale (PMHS) was developed by Lukat, Margraf, Lutz, van der Veld, and Becker [30] to assess mental health status. It consists of a five-point Likert scale ranging from 1 (completely disagree) to 5 (completely agree). Higher scores indicate a more positive mental health status. The PMHS has demonstrated high internal consistency reliability (α = 0.93) [30].

#### 2.3.4. Mental Health Literacy Scale for Healthcare Students

Chao et al. [4] developed a 26-item mental health literacy scale for healthcare students [MHLS-HS] to assess MHL. It consists of a five-point Likert scale ranging from 1 (completely disagree) to 5 (completely agree). Higher scores indicate higher levels of MHL. The MHLS-HS has demonstrated good construct validity (factorial, convergent, discriminant, and known groups validity) (α = 0.81) [4]. It comprises five subscales: maintenance of positive mental health, recognition of mental illness, attitude toward mental illness stigma, help-seeking efficacy, and help-seeking attitude.

The 10-item maintenance of the positive mental health subscale addresses knowledge of competence, autonomy, relatedness, and resilience. It consists of a five-point Likert scale ranging from 1 (completely disagree) to 5 (completely agree). A higher score indicates higher knowledge of positive mental health. The subscale has demonstrated good internal consistency and reliability (α = 0.87) [4]. The four-item recognition of mental illness subscale addresses knowledge about mental illness. It consists of a five-point Likert scale ranging from 1 (completely disagree) to 5 (completely agree). Higher scores indicate more comprehensive knowledge of mental illness. The subscale has demonstrated acceptable internal consistency reliability (α = 0.70) [4]. The six-item mental illness stigma attitude subscale addresses public stigma, dangerousness, and emotional reaction. It consists of a five-point Likert scale ranging from 1 (completely disagree) to 5 (completely agree). All items were reverse scored, and as such, higher scores indicate less problematic attitudes toward mental illness stigma. The subscale has demonstrated acceptable internal consistency reliability (α = 0.76) [4]. The three-item help-seeking efficacy subscale addresses knowledge of when and where to seek professional mental help. It consists of a five-point Likert scale ranging from 1 (completely disagree) to 5 (completely agree). Higher scores indicate more comprehensive help-seeking knowledge. The subscale has demonstrated good internal consistency reliability (α = 0.81) [4]. The three-item help-seeking attitude subscale addresses attitudes toward seeking mental professional help. It consists of a five-point Likert scale ranging from 1 (completely disagree) to 5 (completely agree). Higher scores indicate a greater inclination for help-seeking behavior. The subscale has demonstrated acceptable internal consistency reliability (α = 0.72) [4].

### 2.4. Data Analysis

All statistical analyses were conducted using SPSS version 25 (IBM Corp., Armonk, NY, USA). Frequencies and percentages were calculated for categorical variables, and the means and standard deviations were calculated for continuous variables. A paired sample *t*-test and Cohen’s d test were performed to evaluate the effectiveness of the MHL curriculum intervention. Statistical significance for all analyses was expressed as *p*-value less than 0.05. Cohen’s d of less than 0.2 represents a small effect, about 0.5 represents a moderate effect, and higher than 0.8 represents a large effect [31].

### 2.5. Ethical Procedures

Ethical approval for this study was obtained from local Research Ethics Committees (NTNUREC-201905HS015, No. FJU-IRB NO: C108185). All participants provided written informed consent.

## 3. Results

### 3.1. Descriptive Statistics of Participant Characteristics

As noted, 48 students (11 males and 37 females) completed the intervention. Table 2 provides the characteristics of the participants. The majority of the sample was in the second year of undergraduate studies (97.92%). The mean age was 20.47 years (SD = 0.62), and around 62.50% reported living with their families. The mean score of the Level of Contact Report was 3.52 (SD = 1.76), which was between low and medium (range 2–4). The mean score of the Positive Mental Health Scale was 18.04 (SD = 3.47), which was at high levels (range 15.75–25). The mean scores of MHL and five components at baseline were all high based on the corresponding range of scores.

### 3.2. Effectiveness of the Mental Health Literacy Curriculum

The degree of change between pre- and post-test scores differed significantly (*p* < 0.001): the post-test scores (M = 108.08, SD = 9.30) were higher than the pre-test scores (M = 103.25, SD = 8.38) with a moderate effect size (d = 0.55) (Table 3). To examine the effectiveness of the MHL curriculum on the five components of MHL, the pre- and post-test scores were analyzed. The results showed borderline differences for maintenance of positive mental health (*p* = 0.06) and reducing mental illness stigma (*p* = 0.09). However, compared to the pre-test scores, the relevant scores in both components significantly increased post-test (see Table 3). The post-test scores increased significantly compared to the pre-test scores for recognition of mental illness (*p* < 0.01), help-seeking efficacy (*p* < 0.05), and help-seeking attitude (*p* < 0.05). A small to moderate effect size was found for the recognition of mental illness (d = 0.48), help-seeking efficacy (d = 0.35), and help-seeking attitude (d = 0.39). Significant increases were found for each component of MHL (Table 3).

Regarding the degree of change between the pre-test and follow-up scores, the change in total MHL scores differed significantly (*p* < 0.01): the follow-up scores (M = 108.60, SD = 9.83) were higher than the pre-test scores (M = 103.25, SD = 8.38) with a moderate effect size (d = 0.59) (Table 4). Statistically significant changes were evident in the pre-test and follow-up scores for maintenance of positive mental health (*p* < 0.05) and reducing mental illness stigma (*p* < 0.001). A moderate effect size was found for maintenance of positive mental health (d = 0.49) and reducing mental illness stigma (d = 0.49). There were also significant changes in the items related to both components (see Table 4). In contrast, for recognition of mental illness, help-seeking efficacy, and help-seeking attitude, only small increases were found after the intervention. Compared to the pre-test scores, there was no difference in the follow-up for any item related to the recognition of mental illness. However, there were significant differences for help-seeking efficacy and help-seeking attitude (Table 4).

## 4. Discussion

To the best of our knowledge, this is the first study to examine the effectiveness of MHL interventions among undergraduate public health students. The students’ MHL may have been improved by the educational intervention, which is consistent with prior studies [32,33,34,35]. Research has found that regularly providing an MHL educational curriculum can improve students’ knowledge of mental illness and reduce stigmatizing attitudes significantly and consistently [36]. Atkins, Hoagwood, Kutash, and Seidman [37] and Mcluckie, Kutcher, Wei, and Weaver [38] indicated that school is the most influential environment for students’ cognitive and social development. Therefore, students could improve their MHL by regularly following MHL curricula.

Our MHL intervention had different immediate or delayed effects on each component of MHL. First, we found a significant immediate rather than delayed effect for recognition of mental illness. This indicated that newly acquired knowledge from the intervention might gradually decrease over time, which is in line with previous research [39]. One study showed that the transition from short- to long-term memory requires long-term educational interventions, but long-term intervention is still affected by information overload. Therefore, content related to mental illness should be easy to understand [40]. After the curriculum, information related to mental illness in the mass media and on the Internet may lead to information anxiety among students. An overload of relevant information meant that students cannot determine what is correct [41]. It is suggested that the content of the MHL curriculum be combined to distinguish true and false content regarding mental illness in the mass media or on the Internet in order to reduce information anxiety.

Second, as hypothesized, our study showed that undergraduates’ help-seeking efficacy significantly improved, which is in line with the results of previous studies [3,33]. One study indicated that a school-based MHL intervention effectively improved adolescents’ knowledge of mental illness and help-seeking behavior [33]. A possible reason for this is that interventions at school can provide a complete curriculum, and students can continuously acquire knowledge related to mental health. However, the observed delayed effect decreased over time. A systematic review showed that five facilitators can effectively promote the MHL of adolescents by using interactive teaching methods, supplying diverse educational content, employing trainers with different backgrounds, having direct contact with people with mental illness, and utilizing technological advancements in education [42]. In this study, lecture and cooperative learning were used as teaching strategies for the maintenance of positive mental health, while lectures, contact-based education and cooperative learning were used to improve students’ ability to recognize mental illness, reduce mental illness stigma, increase help-seeking efficacy, and improve help-seeking attitudes. However, only immediate or delayed effects were obtained for each component in this study. Accordingly, it is suggested that a variety of teaching methods (i.e., more than lectures and contact-based education or cooperative learning) be used to increase the effect of such interventions.

Third, the results of this study were consistent with those of others, e.g., that help-seeking attitudes improved significantly after completing the curriculum [43,44]. The literature shows that knowledge of mental illnesses [2], mental illness stigma [45,46,47], and help-seeking efficacy [48] affect help-seeking attitudes. Our MHL curriculum included aspects such as the recognition of mental illness, mental illness stigma, and help-seeking efficacy, which led to significant improvements after the intervention. Accordingly, these findings might account for the increased positive help-seeking attitude.

Fourth, we found a delayed effect in the maintenance of positive mental health. However, research has shown that the positive mental health of adolescents improved immediately after the MEST (MEST is a short version of the Norwegian word for coping) intervention [49]. The intervention aimed to promote positive mental health and well-being among adolescents by inviting them to attend a one-year course. It provided information about understanding personal emotional changes, stress management, relaxation techniques, self-esteem, sleep hygiene, and other related topics [49]. The period of our intervention was shorter than that of the MEST intervention, and the content of our curriculum did not focus solely on the maintenance or improvement of mental health. These factors might have caused the absence of immediate effects in this component of our study. However, the MHL intervention might increase students’ levels of interest in positive mental health and enhance their ability to subsequently assimilate relevant knowledge. This may result in a delayed intervention effect on the maintenance of positive mental health.

Finally, a delayed effect was also found for reducing stigmatizing attitudes toward mental illness. It is noteworthy is that several studies in Western countries showed that destigmatizing interventions had a significant immediate effect on reducing stigmatizing attitudes toward mental illness [50,51,52], or both immediate and delayed effects simultaneously [53]. Prior studies also confirmed culture as one of the factors affecting attitudes toward mental illness. In Asia, a diagnosis of a mental illness is considered shameful. Compared to Western countries, the stigma associated with mental illness is worse in Asian countries [47]. As such, more time may be needed to change attitudes toward mental illness in Asia. To this end, our intervention might increase students’ levels of interest in mental illness, even after the MHL program. This may help students gradually change their attitudes.

This study had several limitations. First, it was conducted using convenience sampling, which limits its generalizability to broader populations. Future research should include more representative samples, e.g., by expanding the intervention to public health students across Taiwan. Second, the sample size calculation was based on a moderate effect size (d = 0.5). The fact that the effect sizes of some components were small might indicate that the sample size was inadequate. Due to the lack of statistical power, the results might be insignificant. Thus, in future studies, the sample size should be increased. Third, the follow-up took place six weeks after the intervention, and a longer effect could not be determined. Future studies could extend the duration of the follow-up to determine whether the results are maintained beyond a six-week period. Finally, the self-reported questionnaire might have led participants to choose options which were inconsistent with their own ideas in order to meet social norms.

## 5. Conclusions

An MHL curriculum may increase the level of MHL among undergraduate public health students. Our findings provide a foundation for the development and implementation of an MHL curriculum for students of public health. Based on this study, we suggest that the program form a mandatory part of the curriculum for public health students and be provided to various students to improve their MHL.

## Figures and Tables

**Table 2:** Characteristics of the sample (*n* = 48).

**Variable**	***n* (%)**	
Gender		
Male	11 (22.92)	
Female	37 (77.08)	
Living situation		
With family	30 (62.50)	
With relatives	1 (2.08)	
With roommates	5 (10.42)	
With friends	8 (16.67)	
Alone	4 (8.33)	
**Variable**	**M (SD)**	**Range**
Age	20.47 (0.62)	18–22
Level of Contact Report	3.52 (1.76)	0–7
Positive Mental Health Scale	18.04 (3.47)	5–25
Mental health literacy (at baseline)	103.25 (8.38)	26–130
Maintenance of positive mental health subscale	41.75 (4.66)	10–50
Recognition of mental illness subscale	17.02 (1.76)	4–20
Reduction of mental illness stigma subscale	21.27 (3.10)	6–30
Help-seeking efficacy subscale	12.15 (1.86)	3–15
Help-seeking attitude subscale	11.06 (2.50)	3–15

**Table 3:** Differences of mental health literacy, subscale and items scores between pre- and post-test surveys (*n* = 48).

Items and Scales	Pre-Test		Post-Test		t	*p*	Effect Size		
	M	SD	M	SD			d	95% C.I.	
Mental health literacy	103.25	8.38	108.08	9.3	4.08	<0.001	0.55	−1.93	3.03
1. Maintenance of positive mental health subscale	41.75	4.66	43.38	4.62	1.9	0.06	0.36	−0.94	1.65
1-1 Handling stressful situations in an appropriate manner.	4.49	0.61	4.65	0.6	1.47	0.15	0.27	0.1	0.44
1-2 Making decisions based on your own will.	3.9	0.95	4.31	0.62	3.52	<0.01	0.52	0.29	0.74
1-3 Setting limits for what is acceptable for you.	4.31	0.95	4.44	0.58	0.8	0.43	0.17	−0.05	0.39
1-4 Having religious or spiritual beliefs.	3.5	0.92	3.67	1.08	1.16	0.25	0.17	−0.11	0.45
1-5 Feeling valuable irrespective of your own accomplishments.	4.38	0.73	4.42	0.79	0.27	0.79	0.05	−0.16	0.27
1-6 Able to adapt to change.	4.55	0.61	4.6	0.57	0.46	0.65	0.09	−0.08	0.25
1-7 Able to achieve goals despite obstacles.	4.15	0.77	4.35	0.64	1.57	0.12	0.29	0.09	0.48
1-8 Able to stay focused under pressure.	3.88	1.1	4.23	0.81	1.92	0.06	0.37	0.1	0.64
1-9 Not easily discouraged by failure.	4.15	0.99	4.19	0.94	0.24	0.81	0.04	−0.23	0.31
1-10 Able to handle unpleasant feelings.	4.46	0.82	4.52	0.71	0.42	0.68	0.08	−0.14	0.29
2. Recognition of mental illness subscale	17.02	1.76	17.83	1.66	3.1	<0.01	0.48	−0.00	0.96
2-1 If someone experiences excessive worry about events.	4.02	0.67	4.46	0.5	3.68	<0.01	0.75	0.59	0.92
2-2 If someone experiences a low mood for two or more weeks.	4.42	0.5	4.56	0.5	1.73	0.09	0.28	0.14	0.42
2-3 If someone requires higher doses of a drug.	4.44	0.68	4.42	0.61	−0.20	0.84	−0.03	−0.21	0.15
2-4 If someone experiences delusions and hallucinations.	4.15	0.65	4.4	0.57	2.59	0.01	0.41	0.24	0.58
3. Reduction of mental illness stigma subscale	21.27	3.1	22.12	3.5	1.76	0.09	0.26	−0.67	1.19
3-1 I think having a mental illness is shameful.	4.29	0.62	4.21	0.71	−0.85	0.4	−0.12	−0.31	0.07
3-2 Most people with mental illness are dangerous.	3.92	0.82	4.08	0.71	1.31	0.2	0.21	0	0.43
3-3 Most people with mental illness are at risk of self-harm.	2.92	0.99	3	1.05	0.41	0.68	0.08	−0.21	0.36
3-4 Most people with mental illness may pose a risk to the public.	3.75	0.86	4.13	0.67	2.77	<0.01	0.5	0.28	0.71
3-5 I think people with mental illness are unpredictable.	2.71	0.99	2.96	1.09	1.55	0.13	0.24	−0.05	0.53
3-6 I think people with mental illness are frightening.	3.69	0.88	3.75	0.84	0.52	0.61	0.07	−0.17	0.31
4. Help-seeking efficacy subscale	12.15	1.86	12.73	1.43	2.36	0.02	0.35	−0.11	0.82
4-1 I know where to go to receive mental health promotion services.	3.77	0.93	4.15	0.58	3.19	<0.01	0.5	0.28	0.71
4-2 I know where to go to receive psychiatry services.	3.98	0.81	4.21	0.5	2.12	0.04	0.35	0.16	0.53
4-3 I know where to obtain information about mental illness.	4.4	0.54	4.38	0.53	−0.24	0.81	−0.04	−0.19	0.11
5. Help-seeking attitude subscale	11.06	2.5	12.02	2.53	2.39	0.02	0.39	−0.32	1.09
5-1 To address a mental health problem, my first choice would be…	4.19	0.7	4.44	0.71	2.21	0.03	0.36	0.16	0.56
5-2 If I face emotional problems, I would seek help.	3.54	1.13	4	1.03	2.44	0.02	0.43	0.13	0.73
5-3 If I were having a mental breakdown, my first inclination would be…	3.33	1.12	3.58	1.13	1.34	0.19	0.22	−0.09	0.54

**Table 4:** Differences of mental health literacy, subscale and items scores between pre-test and follow-up surveys (*n* = 48).

Items and Scales	Pre-Test		Follow-Up		t	*p*	Effect Size		
	M	SD	M	SD			d	95% C.I.	
Mental health literacy	103.25	8.38	108.6	9.83	3.56	<0.01	0.59	−1.96	3.15
1. Maintenance of positive mental health subscale	41.75	4.66	44.06	4.79	2.5	0.02	0.49	−0.83	1.82
1-1 Handling stressful situations in an appropriate manner.	4.49	0.61	4.69	0.59	2.35	0.02	0.34	0.17	0.5
1-2 Making decisions based on your own will.	3.9	0.95	4.33	0.75	3.57	<0.01	0.51	0.27	0.75
1-3 Setting limits for what is acceptable for you.	4.31	0.95	4.58	0.71	1.79	0.08	0.33	0.09	0.56
1-4 Having religious or spiritual beliefs.	3.5	0.92	3.85	0.97	2.51	0.02	0.37	0.11	0.64
1-5 Feeling valuable irrespective of your own accomplishments.	4.38	0.73	4.54	0.65	1.18	0.24	0.23	0.04	0.43
1-6 Able to adapt to change.	4.55	0.61	4.67	0.48	1.01	0.32	0.22	0.07	0.37
1-7 Able to achieve goals despite obstacles.	4.15	0.77	4.35	0.73	1.53	0.13	0.27	0.06	0.48
1-8 Able to stay focused under pressure.	3.88	1.1	4.21	0.82	1.65	0.11	0.34	0.07	0.62
1-9 Not easily discouraged by failure.	4.15	0.99	4.29	0.71	0.91	0.37	0.16	−0.08	0.41
1-10 Able to handle unpleasant feelings.	4.46	0.82	4.54	0.65	0.57	0.57	0.11	−0.1	0.32
2. Recognition of mental illness subscale	17.02	1.76	17.13	2.05	0.33	0.75	0.06	−0.48	0.59
2-1 If someone experiences excessive worry about events.	4.02	0.67	4.25	0.57	1.8	0.08	0.37	0.2	0.55
2-2 If someone experiences a low mood for two or more weeks.	4.42	0.5	4.42	0.58	0	1	0	−0.15	0.15
2-3 If someone requires higher doses of a drug.	4.44	0.68	4.29	0.62	−1.31	0.2	−0.23	−0.42	−0.05
2-4 If someone experiences delusions and hallucinations.	4.15	0.65	4.17	0.66	0.19	0.85	0.03	−0.15	0.21
3. Reduction of mental illness stigma subscale	21.27	3.1	22.88	3.47	3.85	<0.001	0.49	−0.43	1.42
3-1 I think having a mental illness is shameful.	4.29	0.62	4.35	0.67	0.6	0.55	0.09	−0.09	0.27
3-2 Most people with mental illness are dangerous.	3.92	0.82	4.17	0.72	2.21	0.03	0.33	0.11	0.54
3-3 Most people with mental illness are at risk for self-harm.	2.92	0.99	3.13	1.14	1.26	0.22	0.2	−0.1	0.5
3-4 Most people with mental illness may pose a risk to the public.	3.75	0.86	4.1	0.75	2.69	0.01	0.44	0.21	0.66
3-5 I think people with mental illness are unpredictable.	2.71	0.99	3.4	1.05	4.8	<0.001	0.68	0.4	0.97
3-6 I think people with mental illness are frightening.	3.69	0.88	3.73	0.89	0.38	0.71	0.05	−0.2	0.29
4. Help-seeking efficacy subscale	12.15	1.86	12.54	2.71	0.93	0.36	0.17	−0.48	0.82
4-1 I know where to go to receive mental health promotion services.	3.77	0.93	4.15	0.95	2.03	0.05	0.41	0.15	0.67
4-2 I know where to go to receive psychiatry services.	3.98	0.81	4.17	0.93	1.18	0.25	0.22	−0.02	0.46
4-3 I know where to seek information about mental illness.	4.4	0.54	4.23	0.91	−1.24	0.22	−0.23	−0.44	−0.02
5. Help-seeking attitude subscale	11.06	2.5	12	2.65	2	0.05	0.37	−0.35	1.09
5-1 To address a mental health problem, my first choice would be…	4.19	0.7	4.21	0.97	0.13	0.89	0.02	−0.21	0.26
5-2 If I were facing emotional problems, I would seek help.	3.54	1.13	4.04	0.97	2.56	0.01	0.48	0.19	0.77
5-3 If I were having a mental breakdown, my first inclination would be…	3.33	1.12	3.75	1.02	2.25	0.03	0.4	0.1	0.7

## Data Availability

Not applicable.
